# Supplementary material for: Aerosol Delivery of Hesperetin-Loaded Nanoparticles and Immunotherapy Increases Survival in a Murine Lung Cancer Model
Source: Nanomaterials (Basel). 2025 Apr 11;15(8):586. doi: 10.3390/nano15080586 (PMC12029439; doi:10.3390/nano15080586)

Supplementary File.

Title: Aerosol delivery of Hesperetin loaded nanoparticles, and immunotherapy increases survival in a murine lung cancer model

Supplementary Figure 1. Engineering of nanoparticles. a. Apparatus used for microfluidic generation of Hesperetin-loaded nanoparticles. b. Transmission Electron Microscopy (TEM) image of HNP (PEGPLGA-50L) representative of size L.

Supplementary Figure 2. Development of the orthotopic lung tumor model by endotracheal intubation using an endotracheal tumor implant tubing kit for mice. a. Light-guided tubing system for the tumor cell implantation through endotracheal tube to the lung. b. The position of the mice during tumor implantation. Mice were kept in this vertical position for a few minutes to allow the gravitation force to migrate the cancer cells to the lung tissue.

Supplementary Figure 3. Establishment of Aerosol delivery of HNP and Anti-CD40 in Lung Cancer Orthotopic animal model. The picture shows the aerosol drug delivery in mice located in a multi-pocket chamber for treating up to 10 mice at one time connected to a nebulizer system with a drug-mixing chamber for aerosol inhalation.

Supplementary Figure 1.

a

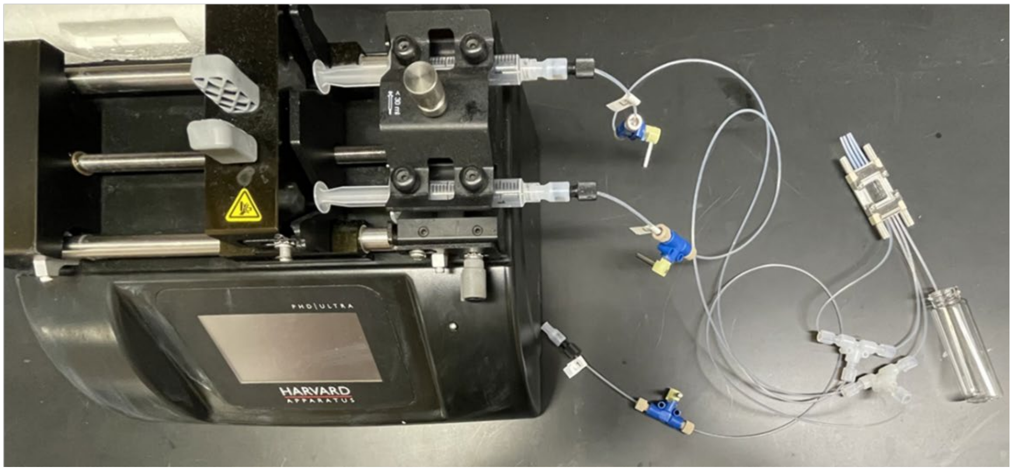

b

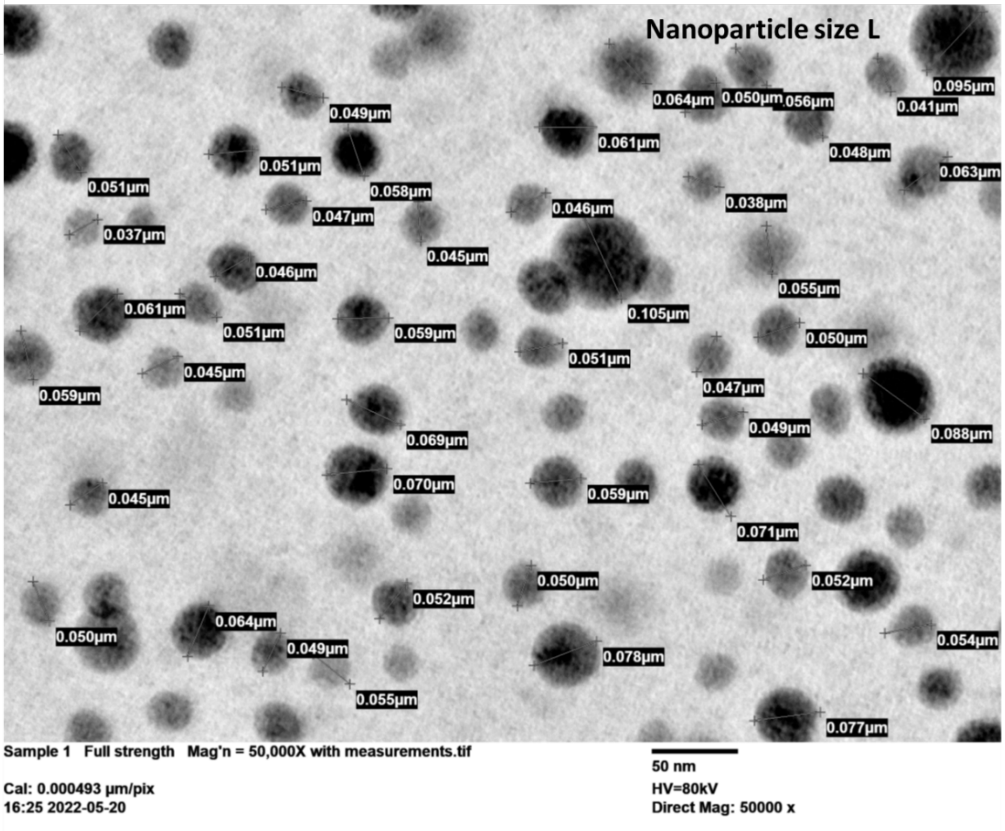

Suplimentary figure 2

a

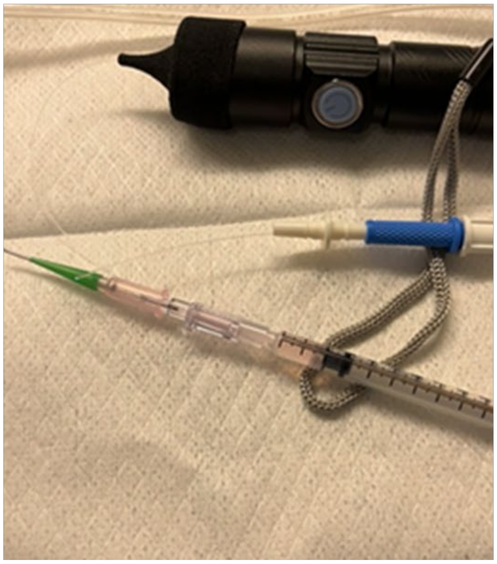

b

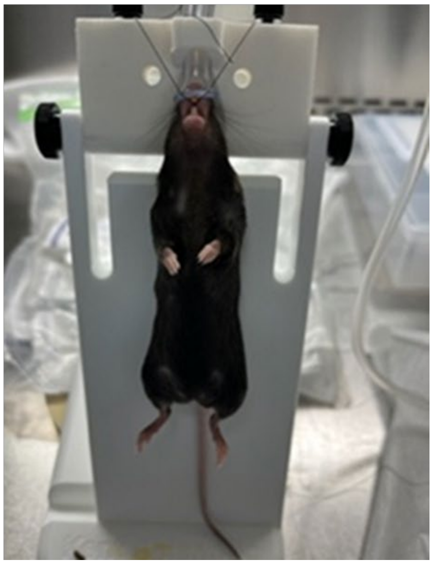

Supplementary figure 3

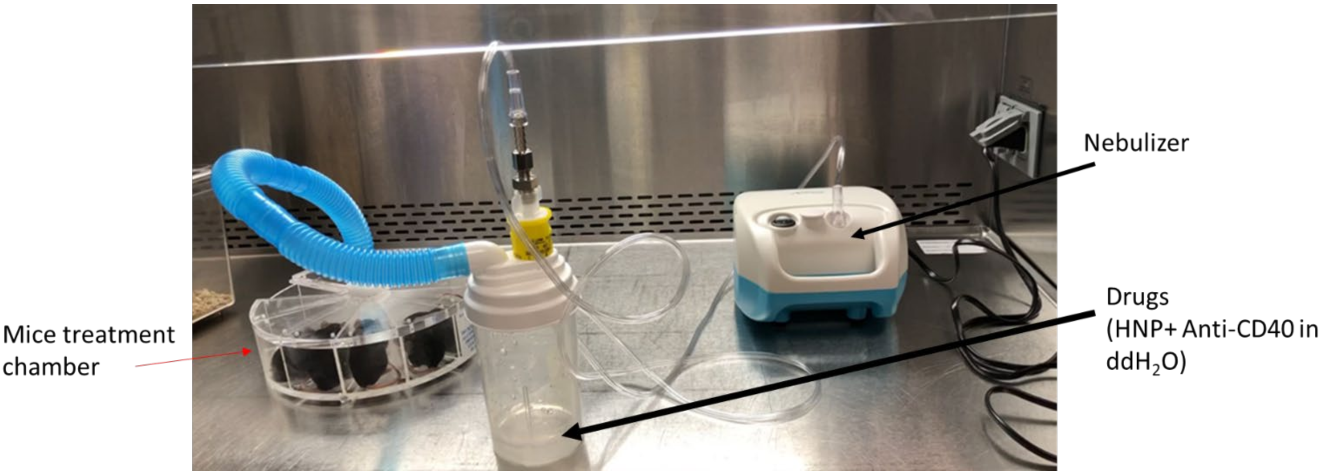

Supplement: Supplementary file 1 [file nanomaterials-15-00586-s001.zip › nanomaterials-3527010-supplementary.pdf]
